# Paroxysmal Nocturnal Hemoglobinuria in the Context of a Myeloproliferative Neoplasm: A Case Report and Review of the Literature

**DOI:** 10.3389/fonc.2021.756589

**Published:** 2021-11-11

**Authors:** Juri Alessandro Giannotta, Bruno Fattizzo, Wilma Barcellini

**Affiliations:** ^1^ Hematology Unit, Fondazione IRCCS Ca’ Granda Ospedale Maggiore Policlinico, Milan, Italy; ^2^ Department of Oncology and Oncohematology, University of Milan, Milan, Italy

**Keywords:** paroxysmal nocturnal hemoglobinuria, myeloproliferative neoplasm, complement inhibitors, thrombosis, bone marrow failure, case report

## Abstract

Paroxysmal nocturnal hemoglobinuria (PNH) is characterized by intravascular hemolytic anemia and thrombosis and is notoriously associated with aplastic anemia and myelodysplastic syndromes. Rarer associations include myeloproliferative neoplasms (MPNs), which are also burdened by increased thrombotic tendency. The therapeutic management of this rare combination has not been defined so far. Here, we describe a 62-year-old man who developed a highly hemolytic PNH more than 10 years after the diagnosis of MPN. The patient started eculizumab, obtaining good control of intravascular hemolysis but without amelioration of transfusion-dependent anemia. Moreover, we performed a review of the literature regarding the clinical and pathogenetic significance of the association of PNH and MPN. The prevalence of PNH clones in MPN patients is about 10%, mostly in association with *JAK2V617F*-positive myelofibrosis. Thrombotic events were a common clinical presentation (35% of subjects), sometimes refractory to combined treatment with cytoreductive agents, anticoagulants, and complement inhibitors. The latter showed only partial effectiveness in controlling hemolytic anemia and, due to the paucity of data, should be taken in consideration after a careful risk/benefit evaluation in this peculiar setting.

## Introduction

Paroxysmal nocturnal hemoglobinuria (PNH) is a rare acquired disorder caused by the somatic mutations of phosphatidylinositol glycan A (*PIGA*). The consequent defect of glycosyl phosphatidylinositol (GPI)-anchored proteins on red blood cell (RBC) surface increases the susceptibility of PNH cells to complement-mediated destruction, leading to intravascular hemolytic anemia, which is the main clinical feature of the disease (1, 2). The natural history of PNH was burdened by high morbidity due to chronic anemia and considerably increased mortality, mainly related to fatal thrombotic events (3). With the advent of complement inhibitors, PNH patients significantly ameliorated their quality of life and survival (4). PNH has been described in the context of bone marrow failure (BMF) syndromes, namely, aplastic anemia (AA) and myelodysplastic syndrome (MDS) (4). However, with the development of more sensitive cytofluorimetric techniques (5), PNH clones of various sizes are increasingly being detected in various onco-hematologic and autoimmune disorders (6–9). The coexistence of PNH and myeloproliferative neoplasms (MPNs) has been reported, but its clinical/prognostic significance and therapeutic management are still poorly known. Moreover, these two conditions share an overlapping clinical presentation, represented by thrombotic events at usual and unusual sites (3, 10–13). Here, we provide the description of a patient with MPN who was subsequently diagnosed with PNH and required specific treatment for hemolytic anemia. In addition, we searched for the available evidence in literature about the association of PNH and MPN, collecting data over the last 40 years in MEDLINE *via* PubMed and the National Library of Medicine. In detail, we reviewed data about the coexistence of clinically overt PNH and MPN, the prevalence of PNH clones in MPN patients, and the prevalence of MPN driver mutations in PNH subjects.

## Case Description

**Figure 1:**
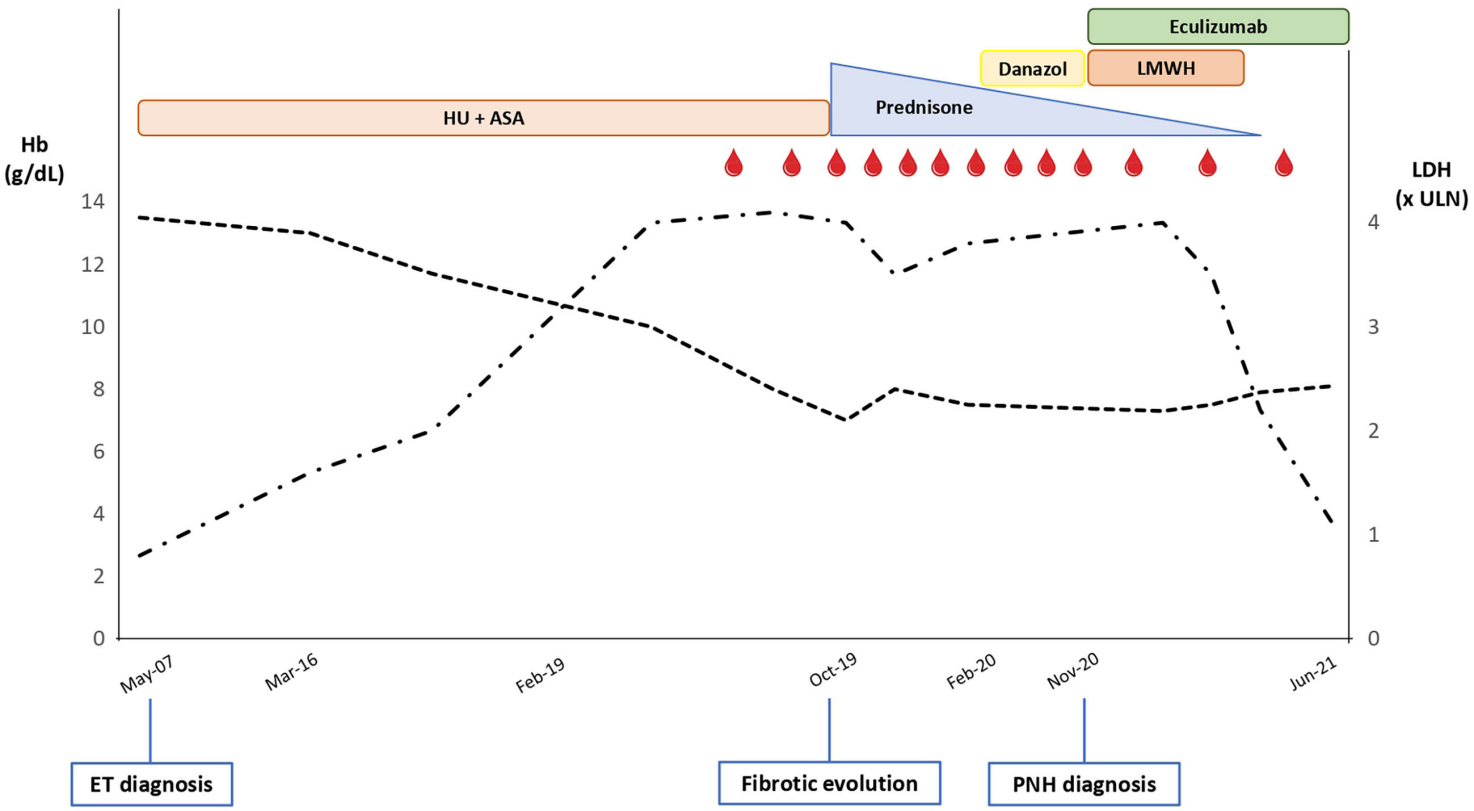
Trend of hemoglobin (Hb; dotted black line) and lactate dehydrogenase (LDH; dashed-dotted black line) levels along the patient’s clinical journey. ULN, upper limit of normal; HU, hydroxyurea; ASA, acetylsalicylic acid; LMWH, low-molecular weight heparin; ET, essential thrombocythemia; PNH, paroxysmal nocturnal hemoglobinuria. Red drops represent red blood cell transfusions.

A 62-year-old Caucasian male was diagnosed with Janus kinase (JAK)2-negative essential thrombocythemia (ET) in May 2007 due to isolated asymptomatic thrombocytosis (**Table 1**). His medical history was unremarkable, except for moderate arterial hypertension on regular treatment; no previous thrombotic events were registered. Bone marrow (BM) evaluation showed normocellularity (40%), increased mature megakaryocytes, slight increase of reticulin fibers (MF-1), and normal karyotype. Once-daily acetylsalicylic acid (ASA) and low-dose hydroxyurea (HU) were started with adequate control of platelet count. From March 2016, a trend to increased lactate dehydrogenase (LDH) levels was noticed, and from February 2019, a mild macrocytic anemia [hemoglobin (Hb) 10.2 g/dL, mean corpuscular volume (MCV) 103 fL; normal iron and vitamin status] developed (**Figure 1**). Direct antiglobulin test (DAT) was negative, with mild elevation of unconjugated bilirubin (UB, 1.3 mg/dL), consumption of haptoglobin, and increased reticulocytes. HU dose was decreased, but anemia worsened (Hb nadir 7 g/dL), LDH rose to 4× upper limit of normal (ULN), and peripheral CD34-positive cells increased. The patient became strongly symptomatic for anemia, requiring about 1–2 RBC units/month. His physical examination was unremarkable. In October 2019, BM reevaluation showed increased cellularity (>95%) with dystrophic megakaryocytes and increased fibrosis (MF-2) and was therefore interpreted as fibrotic evolution of ET. Molecular tests on peripheral blood revealed the presence of a type-2 calreticulin (*CALR*) mutation at exon 9. Abdomen ultrasonography displayed normal spleen and liver size. HU and ASA were stopped, and an attempt with steroids (oral prednisone 50 mg/day) was made with a transient response and reappearance of transfusion dependency during tapering (2–3 packed RBC units/month). In February 2020, danazol was administered, again without anemia improvement. In November 2020, due to persistent intravascular hemolytic anemia and referred dark-colored urines, a flow cytometry for PNH was made and turned positive with a clone size of 95%/94% on neutrophils/monocytes. Low-molecular weight heparin (LMWH) prophylaxis (enoxaparin 4,000 units/day) was started, and the patient was referred to our center for treatment indications. No major PNH-related symptoms were registered. In December 2020, the patient started eculizumab after recommended vaccinations. In the subsequent months, Hb stabilized at 7.5–8 g/dL, LDH progressively lowered to 1.1×–1.2× ULN, and transfusion need returned to 1–2 units/month, with subjective amelioration of the quality of life and disappearance of hemoglobinuria. LMWH was stopped. In June 2021, due to a minor response to eculizumab, a re-evaluation was performed: DAT result turned positive for complement without evidence of cryoagglutinins (as frequently observed under eculizumab treatment), and UB levels slightly increased, indicative of extravascular hemolysis (EVH) during terminal complement inhibitor treatment. BM biopsy confirmed increased age-adjusted cellularity, granulocytic hyperplasia, and numerous megakaryocytes in loose and dense clusters, including both mature and atypical, dystrophic cells; fibrosis was stable with diffuse increase in reticulin and focal bundles of thick collagen fibers (MF-2); of note, hyperplasia of the erythroid lineage in the absence of dysplastic features was described. Karyotype analysis was normal. Analyzed by an expert hemopathologist, the BM trephine was consistent with MPN unclassifiable (MPN-U). A targeted next-generation sequencing (NGS) myeloid panel confirmed the presence of type-2 *CALR* mutation [variant allele frequency (VAF), 45%] and showed an additional somatic mutation in ten-eleven translocation 2 (*TET2*) gene (VAF, 9.7%). The patient is continuing regular fortnightly eculizumab infusions, with subjective benefit, although with persistent transfusion dependence.

**Table 1:** Laboratory parameters at different time points of the clinical history of the patient.

	ET diagnosis (May 2007)	Post-ET MF (Oct 2019)	PNH diagnosis (Nov 2020)	Month +6 after ECU start (June 2021)	Normal ranges
***Hb (g/dL)***	13.5	7*	7.3*	8*	13.5–17.5
***PLT (×10^3^/μL)***	830	396	450	540	130–400
***WBC (×10^3^/μL)***	7.5	4.5	6.0	8.5	4.8–10.8
***LDH (× ULN)***	0.8	4	4	1.1	-
***UB (mg/dL)***	0.5	1.3	1.3	2.0	0–0.8
***Retics (×10^9^/L)***	40	140	200	155	20–100
***Haptoglobin (mg/dL)***	70	<10	<10	<10	30–200
***CD34+ cells (n/μL)***	7	56	56	60	-

ET, essential thrombocythemia; MF, myelofibrosis; PNH, paroxysmal nocturnal hemoglobinuria; ECU, eculizumab; Hb, hemoglobin; PLT, platelet; WBC, white blood cell; LDH, lactate dehydrogenase; ULN, upper limit of normal; UB, unconjugated bilirubin; Retics, reticulocytes. *Pre-transfusion Hb levels.

## Review of the Literature

### Clinical Association of Paroxysmal Nocturnal Hemoglobinuria and Myeloproliferative Neoplasm

Since the 1970s, several case reports about the coexistence of PNH and MPN have been described (**Table 2**). A total of 23 cases have been reported so far, mainly in association with myelofibrosis (MF; n = 12), followed by polycythemia vera (PV; n = 3), MPN-U (n = 2), and only one case each of ET (20) and chronic myeloid leukemia (CML) (19). Nine cases were *JAK2*-positive, while only two *CALR*- and one *MPL*-mutated (24). In 12 cases, the diagnosis of MPN preceded that of PNH, and the clinical suspicion for the latter (when indicated) was the development of hemolytic or iron-deficient anemia (15, 17, 20). The diagnoses were concomitant in seven patients, whose main clinical presentation included atypical thromboses or the coexistence of hemolytic anemia and thrombocytosis (18, 21, 24, 25). In the remaining four subjects, the diagnosis of MPN followed that of PNH and derived from recurrent thrombosis or development of thrombocytosis/leukocytosis (14, 18, 19). Ten patients harbored PNH clones >10% (8/10 were >50%), while in four cases, it was <10%. PNH clone size showed a complessively wide distribution, as reported by Richards et al. (23) in a recent large retrospective study analyzing the clinical presentation of 1,081 PNH patients. The five patients with a previous MPN diagnosis had a PNH clone from 0.7% to 96.3%, while cytopenic/myelodysplastic patients generally harbored smaller clones, and hemolytic/thrombotic subjects harbored larger ones (23). With regard to treatments, eculizumab was administered in four patients (notably, all with *JAK2V617F*-positive MPN). In one patient, eculizumab monotherapy was able to resolve anemia (18); in another, the concomitant administration of eculizumab with acenocumarol and HU resolved a severe case of visceral thrombosis (25). In the remaining two cases, hemolytic anemia was refractory, and one experienced visceral thrombosis recurrence notwithstanding eculizumab plus HU and anticoagulation (18). A retrospective series of 55 PNH patients undergoing hematopoietic stem cell transplantation (HSCT) included two cases associated with MF. The reported overall survival at 5 years was 70%, although the outcome of these two patients is not specified (22). Regarding outcome, four out of the total 23 patients reported (17.4%) died due to acute lymphoblastic/myeloid leukemia progression (16, 17), infections, or liver failure secondary to refractory Budd–Chiari syndrome (18).

**Table 2:** Association of MPNs and PNH.

Type of study, year	N. of patients (sex, age)	Timing of PNH and MPN presentation (delay)	PNH clone size	MPN diagnosis (driver mutation)	Thrombosis	Treatment	Outcome	Notes	Ref.
Case report, 1979	1 (M, 60)	PNH first	-	Not specified	No	Oxymetholone for hypoplastic PNH	-	-	(14)
Retrospective, 1992	47 PNH patients, four of whom with MPN	MPN first	-	PMF	-	-	-	PNH succeeded the development of PMF, while it preceded the diagnosis of MDS.	(15)
Case report, 1993	1 (M, 58)	Concomitant	-	PMF	No	HU, splenic irradiation	Died for AML progression 6 years after diagnosis	AML blasts were PNH+.	(16)
Case report, 2005	1 (M, 53)	MPN first (2 years before PNH)	-	PMF	3 AMI	IFN, HU	Died 6 months after PNH diagnosis due to BCP-ALL progression	PNH diagnosed for iron-deficient anemia.	(17)
Case series, 2012	#1 M, 51 #2 M, 65 #3 M, 78	#1 Concomitant #2 PNH first (2 years before MPN) #3 PNH first (1 year before MPN)	#1 99% G, 13% R #2 40% R #3 73% G, 53% R	#1 and #2 MPN-U (JAK2) *#3* PMF (JAK2)	#1 Multiple thromboses (stroke, BCS) #2 splenic infarction under anticoagulation; BCS under HU and ecu #3 No	#1 Ecu #2 added HU and ecu to anticoagulation #3 ecu, danazol, steroid	#1 Transfusion-free with ecu, but variceal bleeding due to BCS #2 died in 10 months for liver failure and iron overload #3 died for clostridiosis 1 year after	#1 and #2: JAK2V617F detected in PNH+ granulocytes, but not in those PNH- → the JAK2V617F mutation coexists within the PNH clone #3: PMF diagnosed for thrombocytosis; anemia refractory to all treatments.	(18)
Case report, 2015	1 (M, 52)	PNH first (11 years before MPN)	12% CD55-, 24% CD59- G	CML	No	Cyclosporin A, prednisolone, erythropoietin, and Andriol	PNH responsive to IST, CML responsive to imatinib	Disappearance of PNH clones at the time of CML diagnosis.	(19)
Case report, 2016	1	MPN first (6 years before PNH)	73% M, 60% G, 14% R	ET (CALR)	No	No	Alive	Diagnosis of PNH because of hemolytic anemia	(20)
Case report, 2017	#1 F, 72 #2 M, 75	#1 Concomitant #2 MPN first (24 years before PNH)	#1 88.6% G, 86.9% M, 71% R #2 <1%	#1 Post-ET MF (JAK2) #2 PV (JAK2V617F)	#1 Portal vein thrombosis #2 multiple arterial and venous thromboses	#1 HU, anticoagulation #2 anticoagulation, anti-platelets	#1 Recanalization within 2 months #2 Alive	In #1, cell sorting showed that JAK2+ subclone arose within the PNH population.	(21)
Retrospective, 2019	55 PNH patients, two of whom with MPN	Concomitant	-	PMF	-	HSCT	-	Indication for HSCT was association with MF. Complessive 5-year-OS in the cohort: 70%.	(22)
Retrospective, 2020	1081 PNH patients, five of whom with MPN (71, M; 65, F; 55, M; 71, F; 74, M)	MPN first	#1 0.7% #2 93.3% #3 1.2% #4 3.4% #5 96.3%	#1 MF (CALR) #2 not specified (JAK2) #3 PV (JAK2) #4 not specified (JAK2) #5 not specified (JAK2)	#1 DVT/PE #2 BCS	-	-	Severe hemolysis was evident only in Pt #5	(23)
Case report, 2020	1 (M, 49)	Concomitant	99%	PMF (MPL)	No	-	-	The patient presented with anemia, thrombocytosis, elevated LDH, dark-colored urine.	(24)
Case report, 2021	1 (F, 51)	Concomitant	>90%	Masked PV (JAK2V617F)	Venous (hepatic, splenic, kidney)	Anticoagulation, HU, ecu	Clinical resolution of ascites in 2 months	No signs of hemolysis	(25)

MPN, myeloproliferative neoplasm; PNH, paroxysmal nocturnal hemoglobinuria; PMF, primary myelofibrosis; MDS, myelodysplastic syndrome; HU, hydroxyurea; AML, acute myeloid leukemia; AMI, acute myocardial infarction; IFN, interferon; B-ALL, B-acute lymphoblastic leukemia; G, granulocyte; R, red blood cell; MPN-U, MPN unclassifiable; BCS, Budd–Chiari syndrome; ecu, eculizumab; CML, chronic myeloid leukemia; IST, immunosuppressive treatment; M, monocyte; ET, essential thrombocythemia; HSCT, hematopoietic stem cell transplantation; PV, polycythemia vera; DVT, deep vein thrombosis; PE, pulmonary embolism; LDH, lactate dehydrogenase.

### Prevalence of Paroxysmal Nocturnal Hemoglobinuria Clones in Myeloproliferative Neoplasm Patients

Some cross-sectional studies on MPN patients evaluated the association with PNH clones. Tanasi et al. (26) tested 32 patients with MPN and concomitant hemolysis or unexplained anemia and found a PNH clone in three (9.3%) with MF (two *JAK2*- and one *CALR*-mutated). Two of them harbored a PNH clone >90% but did not require specific PNH therapy (26). In a recent large monocentric study, more than 3,000 patients were tested for PNH because of unexplained cytopenia/thrombosis, including 92 patients diagnosed with MPN (27). A PNH clone was found in 16 patients (17.4%), mainly MF, and was generally smaller than 1% (except for one patient with a clone of 5%). It was associated with increased frequency of thrombosis without impact on overall survival (28). In a large cross-sectional study including 197 MPN, 14.2% of subjects had CD55/CD59-negative red cells; this prevalence rose to 21.3% in the subgroup of ET patients (29). At variance, in a series of 98 MPN subjects, PNH clones greater than 1% were detected in only two patients (2%) (30), one of whom with recurrent thrombotic complications. Finally, two studies failed to detect PNH clones in MPN patients. In detail, Nazha et al. (31) tested 62 MF patients with significant anemia (Hb <10 g/dL) and elevated LDH, but none of them harbored a PNH clone. Likewise, a study on 136 patients with myeloid disease, including five MF and 15 MDS/MPN overlap, found GPI-negative cells in 8% of low-risk MDS, but none in MPN subjects (32).

### Prevalence of Myeloproliferative Neoplasm Driver Mutations in Paroxysmal Nocturnal Hemoglobinuria Patients

There are isolated reports of PNH patients harboring mutations in MPN-related driver genes, namely, *JAK2*. Shen et al. (33) performed targeted NGS in a cohort of 36 PNH patients and found *JAK2V617F* homozygous mutations in two patients (5.5%). Langabeer et al. (34) reported a case of AA-PNH with a concomitant *JAK2V617F* mutation at low allele ratio (1.8%) without any clinical feature of MPN; intriguingly, the *JAK2*-positive clone disappeared after cyclosporin therapy, while the PNH clone remained stable. More recently, Santagostino et al. (35) described a case of hemolytic PNH occurring 10 years after HSCT for acute myeloid leukemia (AML); concomitantly, somatic mutation analysis revealed the presence of *JAK2* mutation with an allele ratio of 44% and *TET2* with a VAF of 34%. Soon after, the patient relapsed for AML.

## Discussion

Our patient, along with the others described, allows several clinical and pathogenic considerations about the rare coexistence of PNH and MPN. Besides AA and MDS, PNH clones have been detected also in the context of lymphoid disorders, such as acute lymphoblastic leukemia and lymphomas (9, 36), and in autoimmune/idiopathic cytopenias (6–8, 37). The review of the literature highlighted that about 10% of MPN patients harbor a PNH clone (26, 29, 30), and this frequency rises up to 17% if clones smaller than 1% are considered (27). More importantly, the disregarded association of these two conditions may cause a significant delay in PNH diagnosis, as observed in our case. Additionally, in the MPN setting, the differential diagnosis of hemolytic anemia may be hampered by several confounders: haptoglobin can be decreased in more than 30% of MF (38), LDH is often elevated as a consequence of disease burden (31), and reticulocytosis can be observed in case of myeloid metaplasia (39). Furthermore, the appearance of anemia in MPN should prompt the exclusion of fibrotic evolution, which was in fact observed in our patient, who met the diagnostic criteria for post-ET MF (40). However, at variance with post-ET MF, BM trephine revealed erythroid hyperplasia, which may be attributed to the concomitant peripheral hemolytic process and indicative of bone marrow compensation. Consultation with an expert hemopathologist may be thus advised when morphological findings are not fully consistent with a clear diagnosis and confounding factors coexist.

An important clinical issue is the thrombotic risk in MPN-PNH patients and its management. In our literature review, 35% of MPN-PNH subjects had a severe thrombotic presentation, often refractory to combined anticoagulant, cytoreductive, and anti-complement treatments. This frequency appears higher than that reported for isolated untreated PNH (18.8%) (41) and for MPN (complessively 20%, higher in PV vs. ET/MF and *JAK2*-mutated patients) (12, 13, 42, 43), possibly due to the association of two thrombophilic conditions. With regard to therapy, it is well established that cytoreductive and anti-coagulant/platelet therapies are the cornerstones of thrombosis treatment in MPN, although recurrencies interest about 20% of treated patients (44, 45). In PNH, complement inhibition (Ci) has proven to significantly reduce the thrombotic risk, while anticoagulation alone is poorly effective (4, 46). Whether primary thromboprophylaxis is indicated in untreated PNH is still debated. Given the higher risk observed in patients with a larger clone size (i.e., >50%), they are generally candidates to primary prophylaxis if there are no contraindications. Prophylaxis may be then discontinued once complement inhibitors are started, as in the case described (47). With regard to secondary prophylaxis in patients on complement inhibitors, some experts discontinue anticoagulants when Intravascular hemolysis (IVH) is well controlled by anti-complement therapy (4, 48, 49). Finally, anti-platelet prophylaxis had been stopped in our patient when *CALR*-positive post-ET MF diagnosis was made. In fact, *JAK2*-negative ET is apparently associated with lower rates of thrombosis (50, 51); additionally, the indication to anti-thrombotic primary prophylaxis in MF is not clear-cut (52).

Another relevant clinical issue in MPN-PNH patients may be the infectious risk due to the treatment with anti-complement therapy and the known infectious diathesis observed in MPN (53). Despite the known risk of capsulated bacterial infections under Ci (54), no infectious complications occurred in our patient and in only one out of 23 patients (4%) in the literature (18).

With regard to therapy, in our patient, eculizumab showed effectiveness in controlling IVH but was not able to resolve transfusion-dependent anemia. Accordingly, the review of the literature showed that only a fraction of MPN-PNH patients responded to eculizumab (**Table 2**) (18, 25). In classic PNH, persistent anemia under Ci treatment can be caused by residual IVH, concomitant BMF, and EVH. The management of the latter still remains an unmet need, but promising results are coming from clinical trials with proximal complement inhibitors that avoid deposition of C3 on RBC surface (55).

Many speculations can be made regarding the pathogenic significance of the association of MPN and PNH clones. The evidence of MPN driver mutations, particularly *JAK2*, selectively in GPI-deficient cells (18, 21) has raised the hypothesis that they may confer an intrinsic growth advantage to PNH cells. This cooperative effect has also been proposed for other somatic mutations, such as *TET2* (33, 35, 56, 57), also present in our patient. This view would provide a different explanation to the more common notion that PNH cells have an extrinsic growth advantage secondary to an autoimmune, GPI-selective process against bone marrow precursors (58, 59). Interestingly, MPN diagnosis preceded that of PNH in more than half of cases, and Shen et al. (33) demonstrated that *PIGA* mutation was subclonal to *JAK2*-mutated clone in their report. It is tempting to speculate that the MPN-associated inflammatory microenvironment (60–63) can impair normal hematopoiesis, exerting an “immune pressure” that favors PNH clone expansion, similarly to what happens in AA.

In conclusion, the association of MPN and PNH deserves attention because of the unpredictable clinical course often affected by dramatic thrombotic complications. Since PNH diagnosis is based on highly sensitive and cost-effective techniques, testing for PNH clones should be prompted in any MPN patient showing unexplained anemia with/without frank hemolysis, recurrent thrombosis, and/or atypical BM morphologic findings. Anti-complement treatment in the setting of MPN relies on a careful case-by-case evaluation, weighing the contribution of intravascular hemolysis to anemia and the thrombotic risk.

## Data Availability Statement

The original contributions presented in the study are included in the article/supplementary files. Further inquiries can be directed to the corresponding author.

## Ethics Statement

The studies involving human participants were reviewed and approved by Comitato Etico Milano Area 2. The patients/participants provided their written informed consent to participate in this study.

## Author Contributions

JG, BF, and WB followed the patient, described his clinical history, and revised the literature and the paper for intellectual content. All authors contributed to the article and approved the submitted version.

## Conflict of Interest

WB received consultancy honoraria from Agios, Alexion, Novartis, Annexon, Sanofi, and Sobi. BF received consultancy honoraria from Amgen, Alexion, Novartis, Momenta, Annexon, and Apellis.

The remaining authors declare that the research was conducted in the absence of any commercial or financial relationships that could be construed as a potential conflict of interest.

## Publisher’s Note

All claims expressed in this article are solely those of the authors and do not necessarily represent those of their affiliated organizations, or those of the publisher, the editors and the reviewers. Any product that may be evaluated in this article, or claim that may be made by its manufacturer, is not guaranteed or endorsed by the publisher.
